# RF3:GTP promotes rapid dissociation of the class 1 termination factor

**DOI:** 10.1261/rna.042523.113

**Published:** 2014-05

**Authors:** Kristin S. Koutmou, Megan E. McDonald, Julie L. Brunelle, Rachel Green

**Affiliations:** 1 Howard Hughes Medical Institute, Department of Molecular Biology and Genetics, Johns Hopkins University School of Medicine, Baltimore, Maryland 21205, USA

**Keywords:** ribosome, translation termination, RF3, translational GTPase

## Abstract

Translation termination is promoted by class 1 and class 2 release factors in all domains of life. While the role of the bacterial class 1 factors, RF1 and RF2, in translation termination is well understood, the precise contribution of the bacterial class 2 release factor, RF3, to this process remains less clear. Here, we use a combination of binding assays and pre-steady state kinetics to provide a kinetic and thermodynamic framework for understanding the role of the translational GTPase RF3 in bacterial translation termination. First, we find that GDP and GTP have similar affinities for RF3 and that, on average, the *t*_1/2_ for nucleotide dissociation from the protein is 1–2 min. We further show that RF3:GDPNP, but not RF3:GDP, tightly associates with the ribosome pre- and post-termination complexes. Finally, we use stopped-flow fluorescence to demonstrate that RF3:GTP enhances RF1 dissociation rates by over 500-fold, providing the first direct observation of this step. Importantly, catalytically inactive variants of RF1 are not rapidly dissociated from the ribosome by RF3:GTP, arguing that a rotated state of the ribosome must be sampled for this step to efficiently occur. Together, these data define a more precise role for RF3 in translation termination and provide insights into the function of this family of translational GTPases.

## Abstract (precis)

The precise role of the bacterial class 2 release factor, RF3, in translation termination has been unclear. This study shows that RF3 complexed with GTP but not GDP enhances RF1 dissociation from ribosomes by 500-fold. Importantly, only catalytically active RF1, but not catalytically inactive RF1, is dissociated. This result suggests that a rotated state of the ribosome is required for rapid dissociation.

## INTRODUCTION

Translation termination takes place when ribosomes encounter a stop codon positioned in the decoding center of the small ribosomal subunit. Stop codons are decoded by class 1 release factor proteins (RFs) that act as bifunctional molecules to recognize stop codons with high fidelity and release the growing polypeptide chain in a hydrolytic reaction. Although the bifunctional nature of class 1 RFs is conserved from bacteria to eukaryotes, distinct proteins have evolved in these domains of life to accomplish this task (Youngman et al. 2008). In bacteria, two related proteins, RF1 and RF2, recognize the three stop codons with overlapping specificity (UAA is recognized by both factors, while RF1 is specific for UAG and RF2 for UGA); in eukaryotes, a single factor, eRF1, recognizes all three stop codons.

In addition to the class 1 RFs, another factor (known as a class 2 release factor) is implicated in the termination process. The eukaryotic class 2 factor, eRF3, is functionally essential and universal, while the bacterial class 2 factor, RF3, is nonessential in *Escherichia coli* and not widely distributed throughout the bacterial lineage (Leipe et al. 2002). The molecular basis for the function of class 1 RFs is well defined both biochemically and structurally, but it has been an ongoing challenge to define the molecular role of the class 2 RFs and to rationalize their essential and nonessential natures. Class 2 RFs are translational GTPases that differ substantially in origins and, potentially, function; the sequence of eRF3 is more closely related to the EFTu/eEF1A family of translational GTPases, while the sequence of RF3 more closely resembles EFG/eEF2 (Leipe et al. 2002).

Given the relatedness of RF3 and eRF3 to EFG and EFTu, respectively, we might wonder whether these termination-associated GTPases function in ways consistent with this evolutionary relationship. If this were the case, eRF3 might interact with the nonrotated (classic) state of the ribosome as EFTu does, facilitating the “loading” of the class 1 RF into the A site on termination codons (Schmeing et al. 2009). In contrast, RF3 might interact with the rotated (hybrid) state of the ribosome like EFG, facilitating an event in termination that follows peptide release (Valle et al. 2003; Pulk and Cate 2013; Tourigny et al. 2013; Zhou et al. 2013). Such different potential roles for these factors have, in part, been sorted out using kinetic approaches to define their interactions and actions on the ribosome as outlined below.

In eukaryotes, recent biochemical experiments have shown that eRF3, indeed, plays a role akin to that of EFTu/eEF1A in facilitating the loading of eRF1 into its fully accommodated state in the A site of the ribosome (Eyler et al. 2013). Consistent with this, recent cryoEM data show eRF3 functioning analogously to EFTu, in this case, loading a class 1 RF into the A site (R Beckmann, unpubl.). The essential nature of eRF3 in eukaryotes is consistent with this clear and substantial role for the factor in promoting the process of termination (Kushnirov et al. 1988; Zhouravleva et al. 1995).

Many biochemical studies have similarly contributed to understanding the role of RF3 in bacterial termination. Distinct from the eukaryotic system, RF3 does not enhance the rate of release when RF1 is provided at saturating conditions (i.e., there is no *k*_cat_ effect), but RF3 does increase the overall rate of the release reaction when RF1 is provided at limiting concentrations (Freistroffer et al. 1997; Zavialov et al. 2001). Two potential models could explain such data: (1) RF3 functions to escort the class 1 RF into the ribosome (as eRF3 does for eRF1) (Ito et al. 1996; Nakamura et al. 1996); or (2) RF3 facilitates a post-termination event such as the recycling of the class 1 RF (Goldstein and Caskey 1970). Importantly, further analysis showed that RF3 does not affect the *k*_cat_/*K*_m_ for the release reaction catalyzed by the class 1 RF (Freistroffer et al. 1997), making it unlikely that RF3 plays a role in the initial stages of interaction of the class 1 RF with the ribosome. These data, thus, favor the second model in which RF3 affects events subsequent to the peptide release reaction. These ideas were substantiated by experiments showing that RF3:GTP (and not RF3:GDP) decreases the “recycling time (τ)” for the release reaction (Zavialov et al. 2001). Together, these data suggest that RF3:GTP promotes the dissociation of the class 1 RF from post-release complexes, though this step has not been directly observed.

Another key focus in earlier biochemical studies was on identifying the nucleotide-bound state of RF3 that initially engages the post-release ribosome complex (Zavialov et al. 2001). Other known translational GTPases (EFTu, EFG, and eRF3, for example) engage the ribosome in their GTP-bound state, coupling the energy of GTP hydrolysis to conformational changes in the factor and the ribosome, ultimately leading to factor dissociation. Depending on the off-rate of GDP from the dissociated factor, a specialized GDP-GTP exchange factor (GEF) may be required to reload the GTPase with GTP. In biochemical studies of RF3, Zavialov et al. reported relatively large differences in *K*_D_ for GDP and GTP (5 nM vs. 2500 nM, respectively) and an inherently slow off-rate (*k*_off_) of GDP from the factor. These observations led the authors to propose that a GEF might be required, a prediction supported by the fact that a class 1 RF-programmed ribosome can function to increase the off-rate of GDP from ribosome-bound RF3 (Zavialov et al. 2001). Nevertheless, these studies could not clarify how RF3, liberated of GDP, would efficiently select GTP from solution, given the very large reported differences in *K*_D_.

Structural and biochemical studies provide a working model wherein RF3:GTP first binds to RF1-bound ribosomes found in a nonrotated (classical) conformation and then subsequently promotes the transition of the ribosome into the rotated state, with the concomitant dissociation of the class 1 RF (Ermolenko et al. 2007; Gao et al. 2007; Sternberg et al. 2009; Jin et al. 2011; Zhou et al. 2012b; Pallesen et al. 2013). These observations are broadly consistent with the fact that RF3 is closely related to EFG, which can engage ribosomes in a rotated state to facilitate translocation (Ermolenko et al. 2007; Pulk and Cate 2013; Tourigny et al. 2013; Zhou et al. 2013). Early structural studies had indicated that RF1/2 and RF3:GTP could not bind simultaneously to the rotated state of the ribosome because of steric clashes between the head of the 30S subunit, the 23S rRNA of the 50S subunit, and the class 1 release factor (Gao et al. 2007). Interestingly, a recent cryoEM structure appears to capture a low-occupancy state of the ribosome with both RF3 and RF1 bound (in the nonrotated state); the authors argue that RF3, in this case, has no nucleotide bound (apoRF3) (Pallesen et al. 2013). These observations together correlate nicely with the model that had emerged from kinetic studies invoking increased rates of dissociation of RF1/2 when RF3:GTP engages the ribosome following termination. Importantly, no experiment has ever directly followed the rate of dissociation of RF1/2 from the ribosome, or how this rate is affected by the binding of RF3:GTP.

Here, we present a pre-steady state analysis of the role of RF3 in promoting the dissociation of class 1 RFs following peptide release. Using a reconstituted *E. coli* translation system, we find that the *K*_D_s for GDP and GTP are more closely matched (within fourfold) than previously observed (these data agree with a report published while this manuscript was under review [Peske et al. 2013]). Also, as previously reported (Zavialov et al. 2001; Peske et al. 2013), the off-rates for bound nucleotide (GTP or GDP) are slow, consistent with the need for a GEF to allow translation to proceed at physiological rates. We further show that RF3:GDPNP has considerably higher affinity than RF3:GDP for ribosome termination complexes and that the nonrotated state is slightly preferred. Finally, we use stopped-flow fluorescence approaches to directly follow the dissociation of RF1 from the ribosome upon binding of RF3:GTP and demonstrate that maximal rates of dissociation occur from ribosomes that carry deacylated tRNA in the P site and can thus access the rotated state. These data together refine previous features of the model for RF3 function and allow for a more unified view of the function of translational GTPases.

## RESULTS

### GTP and GDP bind RF3 with similar affinities

**FIGURE 1.**
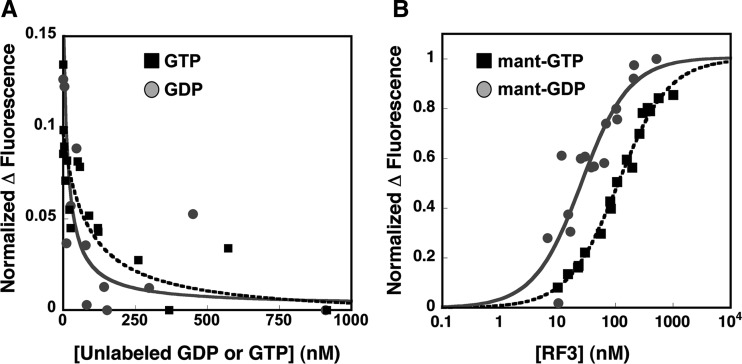
Measurements of nucleotide binding to RF3. Individual mant-GDP data points are displayed in gray circles and mant-GTP data are shown as black squares. (*A*) The change in mant-GDP fluorescence as a function of titrating unlabeled GDP or GTP (0–1000 nM) into solution was monitored on a fluorimeter. The data were fit with equations 1 and 2 to obtain *K*_D_ values. (*B*) The change in fluorescence upon mant-GDP/GTP (5 nM) binding to RF3 (0–1000 nM) was observed on a fluorimeter. These data were fit with equation 3 and the fits are displayed.

To test models related to the nucleotide state in which the GTPase RF3 engages the ribosome, we measured the *K*_D_ of RF3 for GDP and GTP nucleotides in the absence of the ribosome (Fig. 1). We initially attempted to prepare nucleotide-free (apo-) RF3 by incubating RF3 purified from *E. coli* (which copurifies with GDP) (Supplemental Fig. 1) in an EDTA solution for 20 min and then passing the protein through a G-50 sephadex spin-column to remove the unbound nucleotides (Chan et al. 2012). Despite our efforts, this treatment did not remove the bound GDP from RF3 to any significant extent (Supplemental Fig. 1). Other studies have similarly attempted to remove bound nucleotide (Pallesen et al. 2013; Peske et al. 2013). Most recently, Wintermeyer and colleagues demonstrated by FPLC the loss of bound nucleotide but found that RF3 activity was then diminished (Peske et al. 2013). In the studies below, we characterize the binding of functional properties of both naturally isolated RF3:GDP as well as the EDTA-treated RF3:GDP sample and find their properties to be indistinguishable.

Binding constants were determined directly and through competition by fluorescence measurements in buffer containing either HEPES or Tris (50 mM Tris or HEPES at pH 7.6, 70 mM NH_4_Cl, 30 mM KCl, 7 mM MgCl_2_, 5 mM βME). We observed that HEPES altered the fluorescent properties of mant-labeled nucleotides, so the mant-nucleotide binding data reported here were collected in the Tris-containing buffer. For the competition assays, an excess of mant-GDP was incubated with a limiting concentration of RF3 for 30 min to preform RF3:mant-GDP. Increasing amounts of the relevant nucleotide (GDP or GTP) were then titrated into the solution and fluorescence levels determined on a fluorimeter (Fig. 1A). Consistent with the previously published binding affinity of RF3 for GDP (Zavialov et al. 2001; Peske et al. 2013) and the observation that RF3 copurifies with GDP, the *K*_D_ for GDP binding to RF3 is relatively tight (13 nM ± 9 nM) (Fig. 1A). Importantly, however, we find that the affinity of RF3 for GTP is only marginally weaker at 76 nM ± 50 nM (Fig. 1A), at odds with previous studies reporting a *K*_D_ for GTP of ∼2.5 μM (Zavialov et al. 2001), but in agreement with a study published recently (Peske et al. 2013).

**FIGURE 2.**
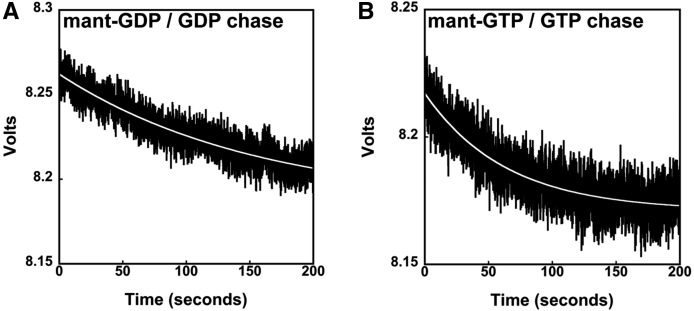
Measurements of the dissociation of mant-nucleotides from RF3. The rate of (*A*) mant-GDP and (*B*) mant-GTP dissociation from RF3 was monitored by stopped-flow fluorescence upon the addition of a nonlabeled nucleotide (GDP or GTP) chase. The data were fit to a single phase, and the fit is displayed as a white line. The rates of nucleotide release from RF3 were slow (*k*_off, mantGDP_ = 0.005 sec^−1^, *k*_off, mantGTP_= 0.01 sec^−1^).

The *K*_D_ values were next measured directly with the fluorescently labeled mant-GDP and mant-GTP derivatives (Fig. 1B). In these experiments, varying levels of RF3 were incubated with limiting mant-GDP and mant-GTP nucleotide for 10 min. Given the measured off-rates of the nucleotides (Fig. 2; Zavialov et al. 2001; Peske et al. 2013), the 10-min incubation is sufficient to allow for exchange with the copurified GDP bound to RF3. Additionally, we note that because RF3 on its own hydrolyzes GTP extremely slowly (<5% is hydrolyzed in 30 min) (data not shown), mant-GTP is unlikely to be hydrolyzed during the course of the binding experiment. These experiments yielded binding affinities for GDP and GTP of 23 nM ± 6 nM and 108 nM ± 8 nM, respectively, closely matching those measured above by competition. These results are summarized in Table 1.

**TABLE 1.**
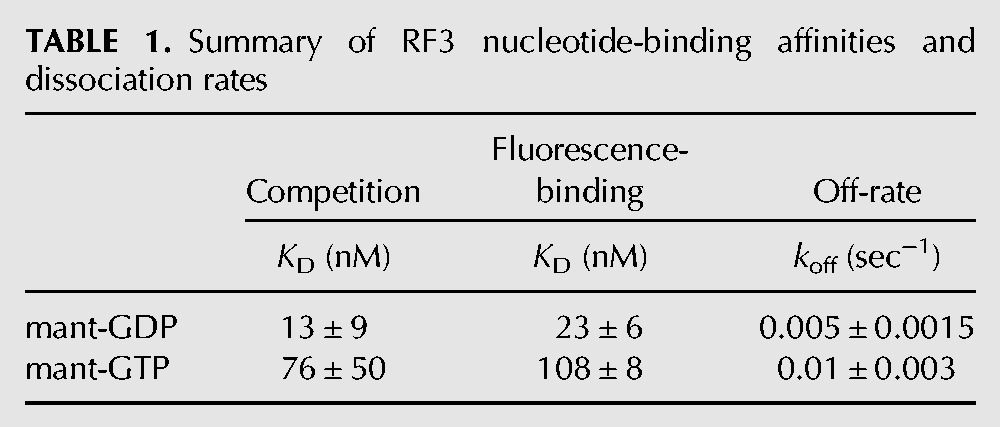
Summary of RF3 nucleotide-binding affinities and dissociation rates

While this manuscript was out for review, Wintermeyer and colleagues published a study that measured the *K*_D_s for RF3 binding to mant-GDP and mant-GTP nucleotides and found them to be 5 and 20 nM, respectively (Peske et al. 2013). Thus, tight RF3-nucleotide (nM) affinities and small (four- to fivefold) preferences for GDP over GTP are consistent across both studies. Given that the in vivo concentration of GTP is approximately eightfold higher than that of GDP (Buckstein et al. 2008), our data suggest that nucleotide-free RF3 in cells will preferentially bind to GTP (and not GDP) in the absence of other contributing factors.

### RF3 releases mant-GDP (and mant-GTP) slowly

While the relative nucleotide-binding affinities of a GTPase for GTP and GDP can be used to determine the likely nucleotide-bound state of a protein in the cell, the kinetic binding parameters (e.g., relative on- and off-rates) must be measured to establish if nucleotide exchange occurs passively or is likely to be facilitated by a GEF. To address potential mechanisms of nucleotide exchange for RF3, we measured the off-rate (*k*_off_) of fluorescently labeled mant-GDP and mant-GTP (in the presence of large amounts of unlabeled GDP or GTP nucleotide as a chase) from RF3 using a stopped-flow apparatus that follows rapid fluorescence changes over time (Fig. 2). The off-rates of mant-GDP and mant-GTP are similar and slow (*k*_off,GDP_ = 0.005 sec^−1^ and *k*_off,GTP_ = 0.01 sec^−1^), suggesting half-lives (*t*_1/2_) for dissociation of nucleotides from the protein on the order of 1–2 min (Table 1). Because these rate constants were relatively slow, we measured them using the same approach but with a fluorimeter, where the apparent change in fluorescence was more dramatic; the same approximate values were obtained (*k*_off,GDP_ = 0.009 sec^−1^ and *k*_off,GTP_ = 0.007 sec^−1^) (Supplemental Fig. 2). These numbers agree well with previous off-rate measurements for GDP (Zavialov et al. 2001; Peske et al. 2013). Together, these data suggest that RF3 may depend on a GEF to promote the rapid exchange of GDP for GTP in vivo, despite their closely matched binding affinities and the excess of GTP in the cell.

### Interactions between RF3:nucleotide and ribosome complexes

#### Pelleting assays with RF3 and ribosomes

**FIGURE 3.**
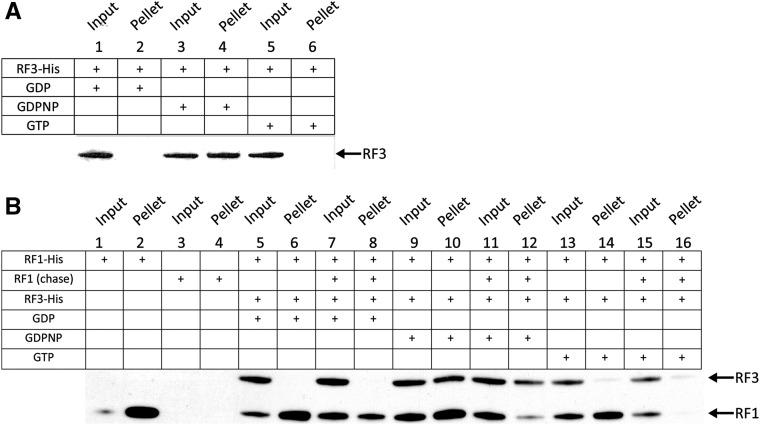
Pelleting assay measuring the binding of RF3-nucleotide to different ribosome complexes. (*A*) Empty 70S ribosomes pelleted following incubation with RF3 bound to GDP, GTP, and GDPNP. Only RF3:GDPNP is detectable after pelleting, suggesting that RF3:GTP can interact with ribosomes in the absence of RF1. (*B*) Termination complexes formed with His-RF1 were pelleted after incubation with RF3 bound to GDP, GTP, and GDPNP in the presence and absence of an unlabeled RF1 chase. These results indicate that RF3:GDPNP and RF3:GTP can promote RF1 dissociation from the ribosome. In all experiments, RF1 and RF3 are His-tagged and their presence detected by Western blot.

We next examined the ability of RF3:GDP, RF3:GDPNP, and RF3:GTP to associate with empty ribosomes as well as programmed “termination” ribosome complexes. We began with a nonequilibrium pelleting assay to determine which forms of RF3 remain stably bound to the ribosome as the solution is pelleted through a sucrose cushion (Pel et al. 1998; Shoemaker and Green 2011). In a first experiment, we evaluated the binding of nucleotide-bound RF3 to empty (unprogrammed) ribosomes by following the presence of RF3 in the pelleted fraction using a His-tagged version of RF3 and Western blot analysis. Here, we found that RF3:GDPNP, but not RF3:GDP and RF3:GTP, bound stably to unprogrammed ribosomes (Fig. 3A).

We next looked at the interaction of His-tagged RF1 and RF3 (bound to the various nucleotides) with ribosome termination complexes where an initiator fMet-tRNA^fMet^ is bound in the P site and the A site is programmed with a stop codon. As with the empty ribosome binding experiments, there is no evidence of stable interaction between RF3:GDP and the ribosome, while RF3:GDPNP is stably bound (Fig. 3B, lanes 6,10); RF3:GTP also appears to bind, albeit considerably more weakly (lane 14). In addition, we note that, in this experiment, RF1 appears to be stably bound to the ribosome even in the presence of RF3:GDPNP or RF3:GTP (Fig. 3B, lanes 10,14). While we cannot strictly establish that RF1 and RF3:GDPNP are bound to the same ribosome, we see incomplete losses in overall RF1 binding in the presence of either RF3:GDPNP or RF3:GTP (Fig. 3B, lanes 2,10,14) and know that termination complexes are formed with >75% efficiency (Zaher and Green 2009).

Because the *K*_D_ for RF1 interacting with ribosome termination complexes is tight (2.5 nM) (Supplemental Fig. 3) and ribosomes were present at 440 nM, we realized that the observed binding could represent either RF1 initially bound to the complexes, or RF1 that rebound following a dissociation event. The latter possibility would explain the observation described above where RF1 appears stably associated with the ribosome in the presence of RF3:GTP (Fig. 3B, lane 14). Current models of translation termination would predict that RF3:GTP promotes RF1 dissociation from the ribosome. To distinguish between these possibilities, we included an RF1 chase (a high concentration of non-His-tagged RF1) with RF3:nucleotide in the binding reaction and followed the binding of His-tagged RF1 and His-tagged RF3 after pelleting. This approach allowed us to determine whether the input RF1 was stably bound during the course of the binding experiments, or if it was undergoing multiple rounds of binding and dissociation. When RF3:GDP is added, there is no substantial decrease in the amount of His-tagged RF1 bound to the release complex (Fig. 3B, lane 8), while His-tagged RF1 binding is substantially diminished in the presence of RF3:GTP and RF3:GDPNP (lanes 12,16). These data are consistent with a model in which the GTP-bound form of RF3 stabilizes a conformational state of the ribosome that promotes RF1 dissociation as previously reported (Gao et al. 2007; Jin et al. 2011; Zhou et al. 2012b; Pallesen et al. 2013).

#### Measuring binding constants for RF3:nucleotide to ribosome termination complexes

**FIGURE 4.**
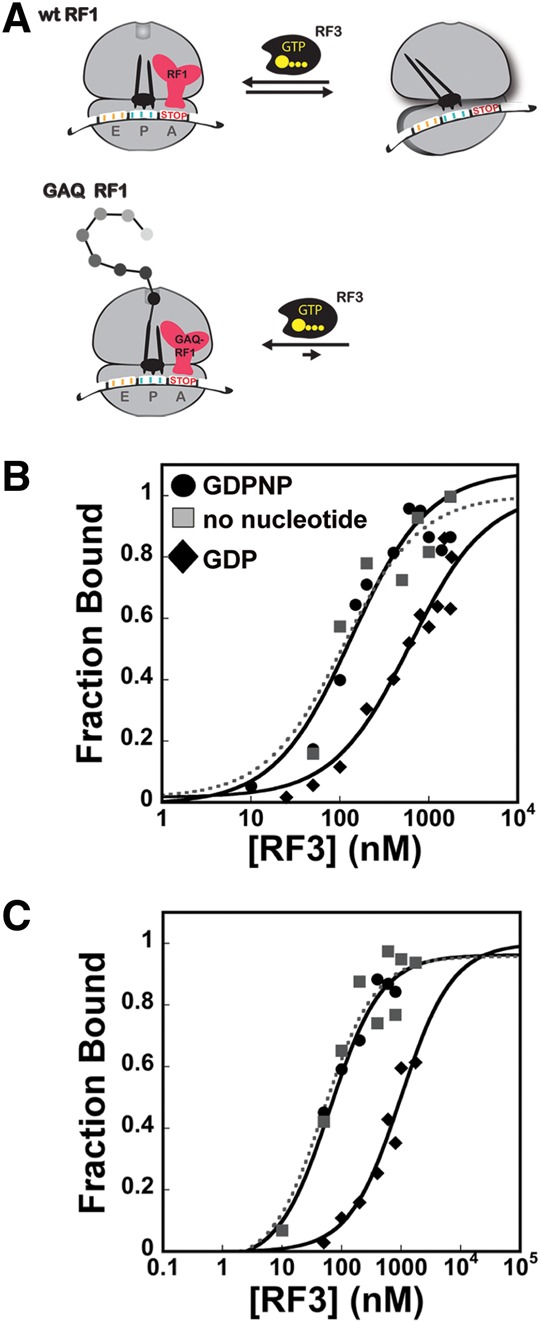
Quantitative filtration assay to determine the binding affinities of RF3-nucleotide for pre- and post-release ribosome termination complexes. (*A*) A representation of the favored ribosome conformations (nonrotated and rotated) in the presence of wild-type and GAQ-RF1. In release complexes containing wild-type RF1, the peptide is released, while in complexes with GAQ-RF1, peptide remains bound and the nonrotated state of the ribosome is favored. (*B*) Binding of RF3 associated with no nucleotide (▪), GDP (♦), or GDPNP (•) to termination complexes formed with wild-type RF1 or (*C*) GAQ-RF1.

While these results provide support for the widely accepted mechanism of action of RF3, we were interested in determining the actual binding affinities of nucleotide-bound RF3 for ribosome termination complexes. In this case, we developed a quick spin gel-filtration assay where the amount of ribosomes and RF3 were determined by Sypro Ruby staining of protein gels run from input and output fractions. As above, stop codon-programmed ribosome complexes were prepared and then treated either with wild-type RF1 or a catalytically inactive variant known as GAQ-RF1 (Zavialov et al. 2002); these variants were prepared as per Shaw and Green (2007). We anticipated that in the presence of wild-type RF1, the peptide mimic (f-Met) would be released from the P-site-bound tRNA and RF1 would be initially bound in the A site and eventually dissociated by RF3:GTP. Ribosomes bound by RF1 (in the absence of RF3) are known by FRET studies to be exclusively found in the nonrotated state (Sternberg et al. 2009). We also performed these studies with the GAQ-RF1 variant, as we anticipated that RF1 would remain bound throughout the experiment, with the ribosome locked in the nonrotated state (Fig. 4A; Moazed and Noller 1989). With this system, we measured the *K*_D_ of RF3 bound to various nucleotides for the different complexes. We see in Figure 4B that RF3:GDPNP binds to the WT RF1 termination complex at least eightfold more tightly than RF3:GDP (50 nM vs. 430 nM *K*_D_s), consistent with our pelleting assay data (Fig. 3B) and with earlier studies (Pel et al. 1998).

Recent cryo-EM studies reported the structure of a post peptide release ribosome containing both nucleotide-free (apo) RF3 and RF1 (Pallesen et al. 2013). This work suggested that, in the presence of substantial concentrations of free GDP, RF3 remains GDP-bound and unable to stably associate with the termination complexes. However, when GDP levels are reduced, the authors argue that RF3 loses stoichiometrically bound GDP and associates with termination complexes in an “apo” state; this loss of GDP could either be spontaneous or facilitated by the ribosome acting as a GEF (Zavialov et al. 2001). The conformation of RF3 in these apo structures differs from those seen for free RF3-GDP (Gao et al. 2007) and for ribosome-bound RF3-GDP(C/N)P (Jin et al. 2011; Zhou et al. 2012b); additionally, the conformation of RF1 is distinct from that observed in ribosomal complexes without RF3 present (Petry et al. 2005; Rawat et al. 2006; Laurberg et al. 2008).

We created a similar situation in our binding assay by adding RF3 (copurified with GDP in 1:1 stoichiometry) to ribosomes complexes with no free GDP or GTP present. Under these conditions, we find that RF3 tightly (*K*_D_ = 36 nM) associates with the ribosome (Fig. 4), likely in an “apo” state. This tight binding is in sharp contrast to the weak association of RF3:GDP (*K*_D_ = 430 nM) for these same complexes. These data suggest that nucleotide-free (apo) RF3 binding to ribosome complexes is more akin to that seen with RF3:GDPNP than with RF3:GDP.

We next measured the association of GDP-, GDPNP-bound, and “nucleotide-free” RF3 with stop codon-programmed complexes reacted with GAQ-RF1. In this situation, ribosomes are thought to be locked in a nonrotated state with variant RF1 bound (Lill et al. 1989; Walker et al. 2008). Here, while the binding trends are the same, we see that RF3:GDPNP binds even more tightly to the ribosome (∼10 nM *K*_D_), while RF3:GDP binds even more weakly (∼1 μM *K*_D_, a 100-fold difference) (Fig. 4C). We note that the *K*_D_ measured with wild-type RF1 is actually a *K*_D,app_ reflecting a combination of the binding constants for multiple conformations of the ribosome (those containing RF1 in a nonrotated state and those not containing RF1 and able to partition between the nonrotated and rotated states), while the *K*_D_ measured with GAQ-RF1 represents the affinity for only the nonrotated conformation of the ribosome with RF1 bound. These data argue that RF3:GDPNP preferentially (and likely, initially) binds termination complexes in the nonrotated (classical) conformation as previously suggested (Sternberg et al. 2009). Furthermore, the ability of RF3:GDPNP to associate with ribosome release complexes containing GAQ-RF1 demonstrates that it is possible for RF3 to bind ribosome release complexes prior to peptide release. However, this observation does not necessarily mean that RF3 binds prior to peptide release, only that it can. These data are all summarized in Table 2.

**TABLE 2.**
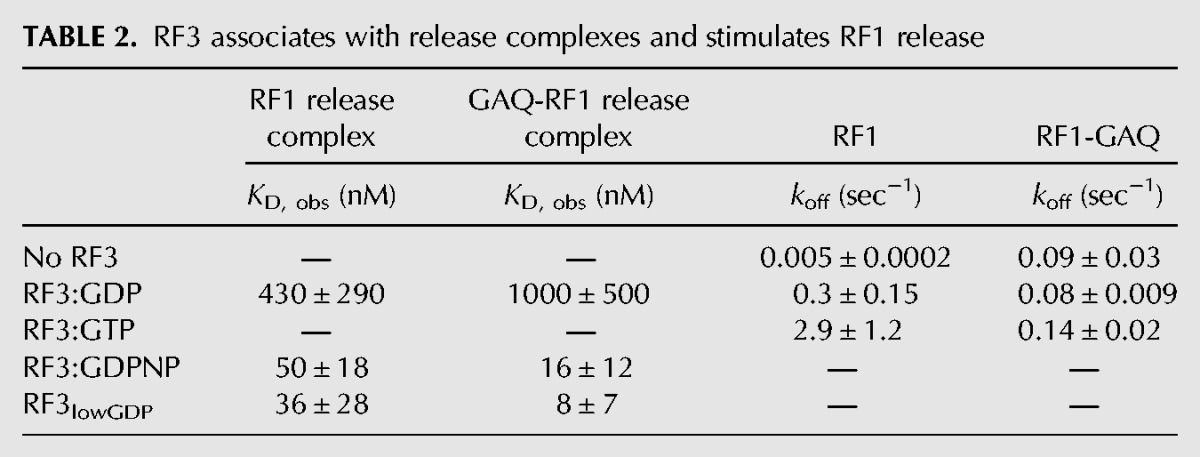
RF3 associates with release complexes and stimulates RF1 release

### RF3:GTP promotes RF1 dissociation

**FIGURE 5.**
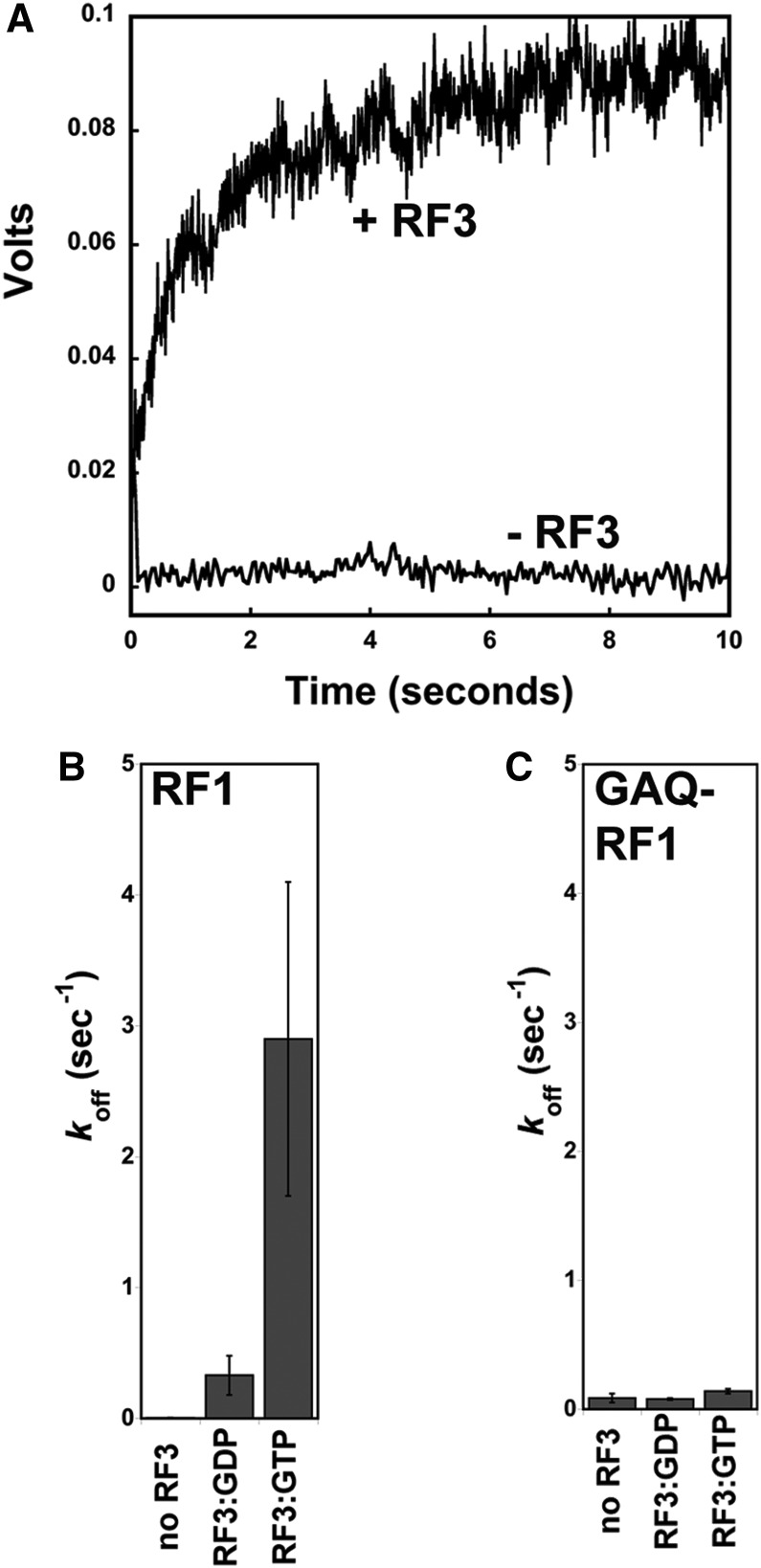
Stopped-flow fluorescent analysis of RF1 dissociation from ribosomes promoted by RF3-nucleotide. (*A*) The dissociation of RF1 from the ribosome in the presence and absence of RF3:GTP as monitored by stopped-flow fluorescence. The rates of RF1 (*B*) and GAQ-RF1 (*C*) dissociation from termination complexes with no RF3, RF3:GDP, and RF3:GTP present are displayed.

In the next step of experiments, we more closely examined RF1 dissociation in the presence and absence of RF3. To monitor RF1 interactions with the ribosome, we prepared a set of single-cysteine variants (A220C and L82C) of RF1 derivatized with fluorescein (Fl-RF1) and determined them to be equivalent to wild-type unmodified RF1 in a peptide release assay (data not shown). We next prepared stop codon-programmed ribosome complexes with the different Fl-RF1 variants and reacted these complexes with RF3 (bound to various nucleotides) in a stopped-flow apparatus. We measured the dissociation rate constant (*k*_off_) for RF1 in the absence of RF3, where Fl-RF1 (either L82C or A220C) was chased off of the ribosome with a 60-fold excess of nonfluorescently labeled RF1. These data were best fit by a double exponential curve. Further experiments revealed the slower rate to be an inherent signal change of RF1, independent of the presence of excess unlabeled RF1, ribosomes, or RF3; as this rate remains constant throughout all experiments, we will not consider it further. Importantly, the rate constant for RF1 dissociation from the ribosome in the absence of RF3 is 0.005 sec^−1^ (Fig. 5A).

We next asked how the addition of saturating levels of RF3 affected the rate of dissociation of RF1. The same complexes were used as above, and now RF3 was added (still with an excess of unlabeled RF1 chase to prevent Fl-RF1 rebinding) with either GDP or GTP (Fig. 5A). Quantitation of the fluorescence traces reveals that RF3:GTP substantially stimulates the rate of dissociation of RF1 from the ribosome by ∼580-fold (stimulated rate of ∼2.9 sec^−1^), while RF3:GDP has a more modest effect (stimulated rate of 0.3 sec^−1^) (Fig. 5B). These data are consistent with our pelleting experiments as well as previous structural and biochemical data arguing that RF3:GTP stabilizes a conformation of the ribosome that promotes the dissociation of the class 1 release factor (Zavialov et al. 2001; Gao et al. 2007; Zhou et al. 2012a).

To further define the mechanistic details of how RF3:GTP promotes RF1 release, stop codon-programmed ribosome complexes were again formed with the catalytically dead GAQ-RF1. The dissociation rate constants (*k*_off_) for GAQ-RF1 were measured as described above for RF1. We first measured the off-rate of GAQ-RF1 in the absence of RF3 and found that GAQ-RF1 is somewhat less stably bound to the ribosome than wild-type RF1 (*k*_off_ = 0.09 sec^−1^ vs. 0.005 sec^−1^) (Fig. 5C). Importantly, however, in the presence of GAQ-RF1, the rate constant for Fl-RF1 dissociation promoted by RF3:GTP is essentially reduced to background levels (*k*_off_ = 0.14 sec^−1^). These results suggest that the rapid release of RF1 from the ribosome depends on the ribosome being able to sample a rotated state. These data are compiled in Table 2.

## DISCUSSION

**FIGURE 6.**
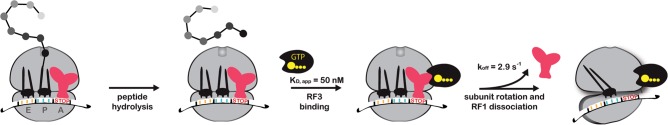
Model for RF3 mechanism of action in translation termination. In this model, following peptide release, with the ribosome in a nonrotated state, RF3:GTP binds the termination complex. The ribosome then undergoes a conformational change to the rotated state, thereby promoting RF1 dissociation.

The data presented here allow us to specify rate and binding constants for several steps in the thermodynamic and kinetic cycle for RF3 in bacterial translation termination (Fig. 6). We first show that RF3 binds GDP with only slightly higher affinity than GTP (13 nM vs. 76 nM, respectively) and that the off-rate for both nucleotides is slow enough that spontaneous dissociation would take, on average, several minutes (Table 1). We note that similar nucleotide-binding and off-rate data were reported by another group after the submission of this manuscript (Peske et al. 2013). While the levels of GTP are eightfold higher than GDP in the cell (1660 μM vs. 233 μM, respectively) (Buckstein et al. 2008), it is not clear how GDP for GTP exchange can happen on a physiologically relevant time scale in the cell in the absence of a GEF that promotes more rapid nucleotide exchange. Indeed, the off-rates of nucleotides from two other translational GTPases, EFTu and EFG, differ greatly from one another: in the case of EFG, the off-rates are relatively fast (*k*_off,GDP_ = 23 sec^−1^ and *k*_off,GTP_ = 7 sec^−1^) (Savelsbergh et al. 2000), while in the case of EFTu, the off-rates are significantly slower (*k*_off,GDP_ = 0.002 sec^−1^, *k*_off,GTP_ = 0.03 sec^−1^) (Gromadski et al. 2002). It is, thus, not surprising that EFTu has a dedicated GEF in the cell, EFTs, while EFG functions as far as is known without any GEF.

In previous studies, the binding constants of RF3 for GDP and GTP were determined to be 5 nM and 2.5 μM (a 500-fold difference), respectively, with an off-rate for GDP of 0.032 sec^−1^ (Zavialov et al. 2001). While our measured off-rate for nucleotide (GDP) closely matches these previous data, our binding affinities for GTP are substantially different (as are those of Peske et al. 2013). Because our data differed from earlier reports (Zavialov et al. 2001), we used two different approaches to confirm the authenticity of our measurements, either directly measuring the affinity for mant-derivatized nucleotide analogs (mant-GDP and mant-GTP) or determining the same parameters in a competition experiment. The overall similarity in these determinations gives us greater confidence in the reported binding data. Taken together, our data suggest that GTP binding should predominate if nucleotide-free RF3 were simply incubated in the cellular milieu; however, given that RF3:GDP leaves the ribosome and must bind to GTP to function in the next cycle, there remains a kinetic problem (Savelsbergh et al. 2000; Gromadski et al. 2002). The off-rates of GDP from RF3 are slow enough that a GEF is likely needed to increase the rate of nucleotide exchange to physiologically relevant levels.

Others have similarly argued that RF3 needs a GEF to promote nucleotide exchange, but for different reasons; these previous studies argued that there are very large differences in *K*_D_ for GTP and GDP, which we find not to be the case (Zavialov et al. 2001). Indeed, the ribosome post-termination complex has been shown in earlier studies to potentially function as the GEF for RF3 (Zavialov et al. 2001; Peske et al. 2013). Our data do not address this claim, and given that this mechanistic feature does not seem to be shared by the other translational GTPases, we remain cautious on this point.

We next established the binding affinities of RF3:GDP, RF3:GDPNP, and likely apo-RF3 for both empty ribosomes and stop codon-programmed ribosome complexes. We used both ribosome pelleting assays and a more quantitative spin-column assay that allowed for relative affinities to be determined. These data were broadly consistent with one another in showing that RF3:GDPNP has overall higher affinity than RF3:GDP for both empty (Pel et al. 1998) and stop codon-programmed ribosome complexes. The affinity of RF3:GDP was ∼10-fold lower for stop codon-programmed ribosome complexes than the affinity of RF3:GDPNP (430 vs. 50 nM, respectively) (Fig. 4B). Moreover, when we prevented sampling of the rotated state by using a GAQ RF1 variant (Zavialov et al. 2002), we saw some strengthening in the binding affinity for RF3:GDPNP and a reduction in affinity for RF3:GDP (10 nM and 1000 nM, respectively) (Fig. 4C). These are ∼100-fold differences in *K*_D_ for these ribosome complexes. Together, our binding and pelleting data suggest that release complexes in the nonrotated state preferentially bind GTP-associated or apo RF3. These data are strikingly consistent with an earlier observation that RF3:GTP can stimulate the *k*_cat_ for peptide release in specialized cases where there is a mismatched codon:anticodon interaction in the P site decoding center (post-peptidyl quality control) (Zaher and Green 2009).

Previous structural studies have consistently failed to capture ribosomes bound to RF3 in a GDP-bound state. Known structures of RF3 bound to the ribosome include X-ray structures of RF3:GDPNP or RF3:GDPCP bound to the rotated, post-termination state of the ribosome (Jin et al. 2011; Zhou et al. 2012b). Previous FRET-based biochemical studies have also clearly shown that RF3:GDPNP stabilizes the rotated state of the ribosome (Ermolenko et al. 2007; Sternberg et al. 2009). Based on our observations (and earlier studies), RF3:GTP likely initially binds to the nonrotated state of the ribosome (possibly even when peptide release has not yet occurred), then stabilizes the rotated state of the ribosome post-peptide release (bringing about RF1/2 dissociation). The idea that RF3:GTP may first bind the nonrotated state of the ribosome is consistent with our pelleting data showing that RF1 and RF3:GDPNP can bind simultaneously to the ribosome (Fig. 3). Additionally, given the similarity in binding behavior of RF3:GDPNP and apo-RF3, our data are also consistent with a recent cryoEM structure of apo-RF3 and RF1 bound to the nonrotated state of the ribosome (Pallesen et al. 2013). While there is no structural data providing a snapshot of RF3:GDP interacting with ribosome termination complexes, the binding data that we provide here offer support for the possible existence of a transient interaction between these components.

In a final set of experiments, we used fluorescently labeled RF1 to directly follow the rates of dissociation of RF1 from termination complexes treated with various nucleotide-bound forms of RF3. While structural and biochemical studies have previously argued that the rotated, RF3-bound state of the ribosome is incompatible with simultaneous binding of RF1 or RF2, the dissociation of class 1 release factors has never been directly observed. First, we show that the rate of spontaneous dissociation of wild-type RF1 from ribosome termination complexes is slow (0.005 sec^−1^); the GAQ RF1 variant is somewhat less stably bound, with a spontaneous dissociation rate of 0.09 sec^−1^. Next, we demonstrate that addition of RF3:GTP dramatically increases the rate of dissociation of RF1 from the ribosome (by ∼580-fold); this stimulation is larger than previously predicted based on multiple turnover kinetics (Zavialov et al. 2001). Importantly, this stimulatory effect is wholly abrogated when the GAQ RF1 variant is instead used to form the termination complexes. These data are consistent with the idea that movement of the ribosomal subunits into the rotated state is essential for promoting rapid dissociation of RF1 from the complex. These data provide the first direct observation of the RF1 departure as stimulated by RF3:GTP and give some sense of the extent to which RF3 enhances RF1 dissociation.

In closing, it is worth considering that RF3 is a nonessential gene in *E. coli* (Mikuni et al. 1994) and is not conserved across the bacterial lineage (Margus et al. 2007). Given the very substantial stimulation of the dissociation of RF1 that is promoted by RF3 (∼580-fold), it seems surprising that such a role would not be essential, unless this step is not rate-limiting for the overall process. Alternatively, in the cell, other factors may function redundantly with RF3 in promoting class 1 termination factor dissociation; the recycling factors RRF and EFG come to mind as potentially substituting for RF3 in this role (Zavialov et al. 2005).

## MATERIALS AND METHODS

### Purification of release factors

His-tagged RF3 was overexpressed and purified in BL21 DE3 pLysS cells as previously described (Zaher and Green 2009). To try to remove copurified GDP from RF3, protein was incubated in Buffer 219_HEPES_ (50 mM HEPES at pH 7.6, 70 mM NH_4_Cl, 30 mM KCl, 7 mM MgCl_2_, 5 mM βME) plus 30 mM EDTA at 26°C for 20 min to chelate all magnesium and release GDP from RF3. Samples were then passed over a Sephadex G-50 (cutoff > 30 kD; RF3 is 60 kD) column pre-equilibrated with Buffer 219 + 30 mM EDTA at 6000*g* for 15 sec to separate nucleotide-free RF3 from GDP in solution. Flow-through was collected and MgCl_2_ added to return the sample to overall 7 mM MgCl_2_ in solution. EDTA-treated RF3 was then aliquoted, flash frozen, and stored at −80°C.

Through site-directed mutagenesis, the three native cysteines in RF1 were removed by mutation to serine, and new cysteines were introduced at several positions (A220C and L82C) based on solvent accessibility, sequence conservation, and proximity to areas of interest in RF1. Mutant N-terminally His-tagged RF1 was expressed and purified from pET15b-RF1 in BL21 DE3 pLysS cells as previously described (Shimizu et al. 2001). RF1 was buffer exchanged into Labeling Buffer (20 mM HEPES pH 6.8, 7 mM MgCl_2_, 0.2 mM TCEP) and incubated with 10-fold excess fluorescein-maleimide for 2 h at room temperature, then overnight at 4°C. Excess dye was removed by nickel chromatography. Labeled RF1 was dialyzed against RF storage buffer (30 mM Tris-HCl pH 7.5, 70 mM NH_4_Cl, 30 mM KCl, 7 mM MgCl_2_, 5 mM β–mercaptoethanol, 50% glycerol) and stored at −20°C. The GAQ mutation was incorporated into the single cysteine L82C and A220C RF1 mutants generated above, and labeled and purified as described for catalytically active RF1.

### Detecting nucleotide bound to RF3 by HPLC

An HPLC assay was used to detect nucleotide (GDP and GTP) bound to RF3, and EDTA-treated RF3 (Supplemental Fig. 1). Protein samples were prepared first making a 71.05 μL solution containing: 60 μM protein sample (or GDP/GTP in control samples), 5 μL 219_Tris_ Mg(II)-free buffer, 0.6 μL 70% perchloric acid. The solution was incubated at 22°C for 2 min to denature protein, and the pH subsequently adjusted with 3.95 μL 3 M NaOAc, pH 5.1. The sample was then spun for two minutes in a bench-top microfuge to pellet protein. The supernatant containing nucleotide was removed from the pellet and diluted (1:1) with 75 μL of Solvent A (100 mM KH_2_PO_4_ pH 6.5, 10 mM tetrabutylammonium hydrogen sulfate) to yield a final volume of 150 μL. The sample was then filtered through a 0.22-μm syringe filter to remove any magnesium precipitate.

HPLC (Agilent 1200) was used to assess the nucleotide content of the samples in a protocol adapted from Smith and Rittinger (2002) and described below. The sample was injected onto an Agilent Zorbax Eclipse XDB-C18 (4.6 × 150 mm, 5 µ) column using 150 μL inserts for 2-mL HPLC vials, and eluted on a gradient with a 1 mL/min flow rate. The two solvents used were Solvent A (above) and Solvent B (100 mM KH_2_PO_4_ pH 6.5, 10 mM tetrabutylammonium hydrogen sulfate, 30% acetonitrile). The gradient was run as is noted in Table 3.

**TABLE 3.**
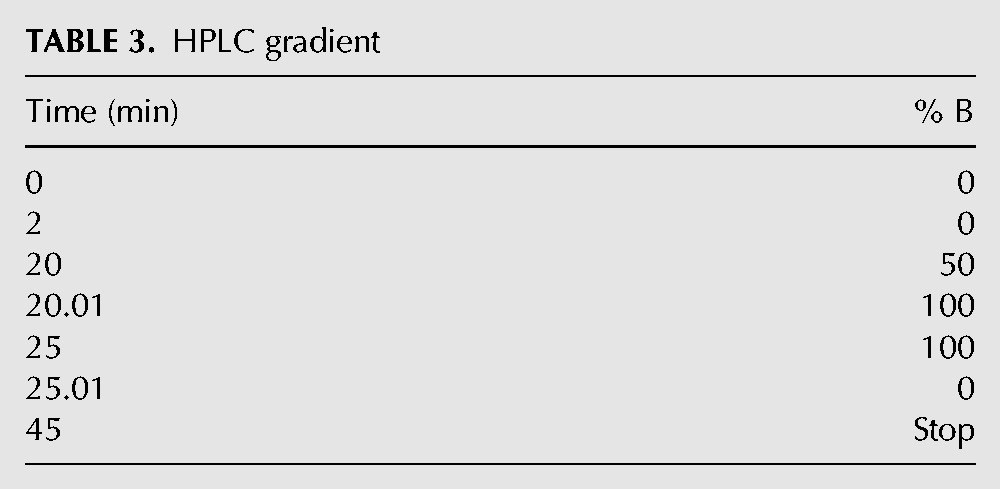
HPLC gradient

### Mant-nucleotide binding to RF3

#### Competing off mant-GDP with unlabeled GTP/GDP

For our competition assays, an excess of mant-GDP (200 nM) was incubated with a limiting concentration RF3 (10–15 nM) for 30 min to form a RF3:mant-GDP complex. Increasing amounts of the relevant nucleotide (unlabeled GDP or GTP) were titrated in and fluorescence levels determined on a fluorimeter (Fig. 1). The data were fit with two equations (Equations 1 and 2). Equation 1 assumes that the *K*_D_ of both mant-GDP and the unlabeled nucleotides is unknown, and Equation 2 assumes that the *K*_D_ of mant-GDP is ∼5 nM (Zavialov et al. 2001; Peske et al. 2013). Both fits yielded approximately equivalent binding constants for GDP and GTP.

Equation 1:

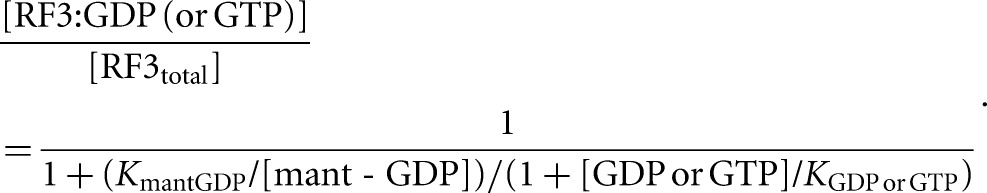

Equation 2 (Zavialov et al. 2001):

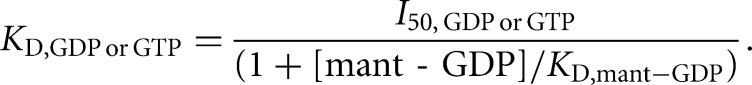



#### Binding of mant-nucleotides

Fluorescence experiments were performed on a Fluorolog-3 spectrofluorometer (Horiba Jobin Yvon). Five nanomoles mant-GDP or mant-GDPNP (Invitrogen) were incubated for 10 min in Buffer 219_Tris_ (50 mM Tris at pH 7.6, 70 mM NH_4_Cl, 30 mM KCl, 7 mM MgCl_2_, 5 mM βME) with increasing concentrations of nucleotide-free RF3, from 0 nM to 1000 nM, at 26°C. An excitation wavelength of 355 nm was used, and emission spectra were gathered between 400 and 500 nm (emission maximum near 448 nm). Photobleaching of mant-nucleotide was negligible over the course of the experiment. The volume of the overall reaction through the course of RF3 titration did not change more than 10%. Peak fluorescence values were measured at the emission maximum (440 nm for these experiments) and fit with a quadratic binding isotherm (Equation 3):

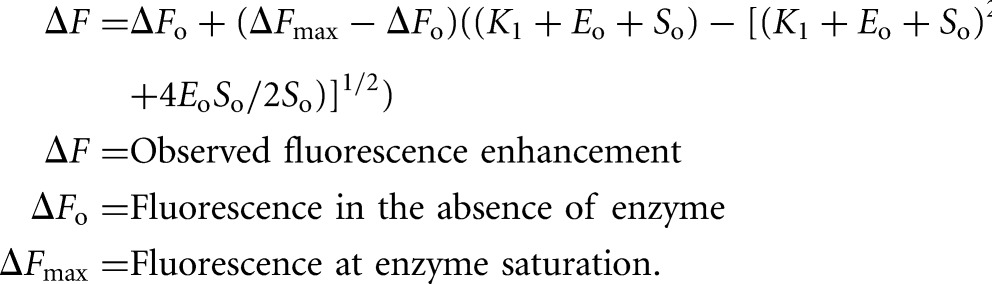



### Mant-nucleotide dissociation from RF3

To measure the rate of mant-nucleotide dissociation from RF3, an excess of apo-RF3 was preincubated with a limiting amount of mant-nucleotide in 1X buffer 219_Tris_ at 26°C for 10 min in order to form the RF3:nucleotide complex. Mant-nucleotides were subsequently chased off of RF3 with a large excess (1–4 mM) of unlabeled (“cold”) GDP or GTP. The change in fluorescence upon the addition of “cold” nucleotide was monitored as a function of time either by stopped-flow or fluorimeter. In the case of the stopped-flow experiments, equal volumes (50 µL each) of the reactants (2 µM apo-RF3 and 40 nM mant-nucleotide in one syringe, and 4 mM “cold” nucleotide chase in a second syringe) were rapidly mixed. For the fluorimeter-based assay, 3.03 μL of 100 mM GDP or GTP was added to 300 μL of RF3:mant-nucleotide complex formed with 200 nM RF3 and 5 nM mant-nucleotide, and mixing was accomplished manually (via pipette). The resulting fluorescence traces from both experimental set-ups were fit to the following equation (Equation 4): *F*_o_ + Δ*F* × e^−kt^ (*F*_o_ = initial fluorescence at time 0, Δ*F* = change in fluorescence/amplitude, and *k* = *k*_off_), using Kaleidagraph software.

### Pelleting assay with Western blot

Reactions were incubated in Buffer 219_HEPES_ (50 mM HEPES at pH 7.6, 70 mM NH_4_Cl, 30 mM KCl, 7 mM MgCl_2_, 5 mM βME) containing 0.4 μM empty 70S ribosomes (containing no mRNA or tRNAs), or 0.4 μM ribosome release complex (on fMet-STOP mRNA) preincubated with 1 μM His-tagged RF1 and 1 μM His-tagged RF3 for 10 min at 37°C. Ribosome termination complexes (composed of 70S ribosomes, fMet-STOP mRNA and fMet-tRNA^fMet^) were formed with an efficiency >75% by following previous protocols (Zaher and Green 2009). Nucleotide-free RF3 was preincubated with either 1 mM GDP, 1 mM GDPNP, or 1 mM GTP for 10 min at 37°C prior to addition. Reactions were pelleted through 600 μL Buffer D (1.1 M sucrose, 20 mM Tris-HCl pH 7.5, 500 mM NH_4_Cl, 10 mM MgCl_2_, 0.5 mM EDTA) for 2 h at 70,000 rpm in a TLA-100.3 rotor. The resulting pellets were resuspended in 1X Buffer 219_HEPES_ and analyzed via Western blot analysis with Penta-His HRP-conjugated antibody (Qiagen). Non-His-tagged RF1 was prepared by adding TEV protease to His-tagged RF1 to a final TEV concentration of 50 μg/mL and incubating the mixture at room temperature for 4 h. The cleaved protein was then passed over a Ni-NTA column to collect the flow-through. This removes the TEV protease and the cleaved His-tag, and the flow-through containing RF1 was dialyzed into storage buffer (30 mM Tris-HCl pH 7.5, 70 mM NH_4_Cl, 30 mM KCl, 7 mM MgCl_2_, 5 mM β–mercaptoethanol, 50% glycerol).

### Measuring the binding of Fl-RF1 to ribosomes

The affinity of RF1 for ribosomes on an AUG-UAA message programmed with ^f^methionine in the P-site was measured using fluorescence titrations. In these reactions, 1 nM of fluorescently labeled A220C RF1 was titrated with ribosome complexes (0–52 nM final concentration) in 1X 219-HEPES buffer. The fluorescence intensities (or counts) (λ_excitation_ = 491 nm, λ_emission_ = 515 nm) were plotted as a function of ribosome concentration and fit as described above in Equation 3.

### Measurement of K_D_ for RF3 to release complex

Prepacked columns were prepared as follows: Sephadex G-100 (GE Healthcare Life Sciences; cutoff > 100 kD) was swelled in water, then buffer-exchanged into Buffer 219_HEPES_ (50 mM HEPES at pH 7.6, 70 mM NH_4_Cl, 30 mM KCl, 7 mM MgCl_2_, 5 mM βME) immediately prior to the experiment. Eight hundred microliters of 50–50 slurry were applied to a Corning Costar Spin-X centrifuge tube filter (Sigma-Aldrich) and centrifuged at 3000*g* for 20 sec. Flow-through was removed, and the column was used in the reaction below.

One hundred fifty nanomoles nucleotide-free RF3 were incubated with 2 mM GDP or GDPNP—in the case of the “apo” assay, no nucleotide was added, but RF3 was still subjected to same incubation time—for 10 min at 26°C. Preprogrammed STOP complexes in concentrations ranging from 10 nM to 2 µM were incubated with threefold excess RF1 in Buffer 219 to ensure a fully formed release complex. Equal volumes (20 µL) of the reactants were then mixed and incubated for 10 min at 37°C. Each reaction mixture was loaded onto a prepacked Sephadex G-100 column as prepared above, and unbound RF3 (60 kD) was separated from the release complex by centrifugational gel filtration at 3000*g* for 20 sec (Koutmou et al. 2011).

Flow-through was collected and the total volume recorded, then 35 µL of each reaction were loaded onto a Criterion XT 4%–12% Bis-Tris gel (Bio-Rad) and run for 1 h at 15 W. Gels were stained with the quantitative dye SYPRO Ruby (Invitrogen) for at least 3 h for maximum sensitivity and destained with a solution of 10% methanol and 7% acetic acid for 30 min. Gels were quantitated by a PhosphorImager and analyzed using ImageQuant TL (GE Life Sciences). Amounts of RF3 bound to ribosome (normalized for flow-through volume) were graphed vs. ribosome concentration, and data were fit with a quadratic function to determine *K*_D_.

### Measurement of rate of dissociation of fluorescent-RF1 from ribosome

Fluorescence stopped-flow measurements were performed on an SX-20 spectrometer (Applied Photophysics) in Buffer 219_Tris_ (50 mM Tris at pH 7.6, 70 mM NH_4_Cl, 30 mM KCl, 7 mM MgCl_2_, 5 mM βME) at 26°C. The interaction between RF1 on the release complex and RF3 was monitored by change in fluorescein fluorescence. Reactions were prepared as follows: RF1 proteins were fluorescein-labeled at positions A220C and L82C as described above in functional RF1 as well as the catalytically incompetent GAQ mutant RF1. One hundred fifty nanomoles ribosome complex were preincubated with 75 nM fluorescein-RF1 for 10 min at 26°C. One micromole nucleotide-free RF3 was preincubated with 1 mM GDP or GTP for 10 min at 26°C. Unlabeled RF1 was also added to the RF3 reactant mixture as a “cold” chase at a final concentration of 1 μM to prevent rebinding of the fluorescein-RF1 to release complexes. A functional RF1 was used as the chase for the GAQ mutant experiment as well.

Experiments were performed by rapidly mixing equal volumes (50 µL each) of the reactants (release complex + fluorescein-RF1 in one syringe, and RF3 + nucleotide + unlabeled RF1 chase in the second) and measuring the time course of fluorescence change. Data depicted in Figure 5 were collected with a sample rate every 20 msec with the use of oversampling to average every 5–10 transient samples. Data were evaluated by fitting to a biphasic exponential function yielding two rates [(Δ*F*_1_ × e^−k1t^) + (Δ*F*_2_ × e^−k2t^) + *F*_o_] (Equation 4), where Δ*F* = amplitude of the phase, *k* = rate, *t* = time, and *F*_0_ = fluorescence at time 0, using Kaleidagraph software.

## SUPPLEMENTAL MATERIAL

Supplemental material is available for this article and contains data demonstrating that GDP copurifies with RF3 and cannot be removed by a simple EDTA treatment (Supplemental Fig. 1), the mant-GDP and mant-GTP off-rates from RF3 are slow as measured on a fluorimeter (Supplemental Fig. 2), and the affinity of Fl-RF1 for stop-codon programmed ribosome complexes is tight (Supplemental Fig. 3).
